# Interplay between LHCSR proteins and state transitions governs the NPQ response in *Chlamydomonas* during light fluctuations

**DOI:** 10.1111/pce.14372

**Published:** 2022-06-21

**Authors:** Collin J. Steen, Adrien Burlacot, Audrey H. Short, Krishna K. Niyogi, Graham R. Fleming

**Affiliations:** ^1^ Department of Chemistry University of California Berkeley California USA; ^2^ Molecular Biophysics and Integrated Bioimaging Division Lawrence Berkeley National Laboratory Berkeley California USA; ^3^ Kavli Energy Nanoscience Institute Berkeley California USA; ^4^ Howard Hughes Medical Institute University of California Berkeley California USA; ^5^ Department of Plant and Microbial Biology University of California Berkeley California USA; ^6^ Department of Plant Biology Carnegie Institution for Science Stanford California USA; ^7^ Graduate Group in Biophysics University of California Berkeley California USA

**Keywords:** bioenergetics, chlorophyll fluorescence, light‐harvesting complex stress related, microalgae, nonphotochemical quenching, photoprotection

## Abstract

Photosynthetic organisms use sunlight as the primary energy source to fix CO_2_. However, in nature, light energy is highly variable, reaching levels of saturation for periods ranging from milliseconds to hours. In the green microalga *Chlamydomonas reinhardtii*, safe dissipation of excess light energy by nonphotochemical quenching (NPQ) is mediated by light‐harvesting complex stress‐related (LHCSR) proteins and redistribution of light‐harvesting antennae between the photosystems (state transition). Although each component underlying NPQ has been documented, their relative contributions to NPQ under fluctuating light conditions remain unknown. Here, by monitoring NPQ in intact cells throughout high light/dark cycles of various illumination periods, we find that the dynamics of NPQ depend on the timescales of light fluctuations. We show that LHCSRs play a major role during the light phases of light fluctuations and describe their role in growth under rapid light fluctuations. We further reveal an activation of NPQ during the dark phases of all high light/dark cycles and show that this phenomenon arises from state transition. Finally, we show that LHCSRs and state transition synergistically cooperate to enable NPQ response during light fluctuations. These results highlight the dynamic functioning of photoprotection under light fluctuations and open a new way to systematically characterize the photosynthetic response to an ever‐changing light environment.

## Summary Statement

Plants and algae must dynamically acclimate to the unpredictable fluctuating light conditions found in the natural environment. Here we show that in a green microalga, the dynamics of photoprotection depend on the timescale of light fluctuations and reveal that light‐harvesting complex stress‐related (LHCSR) proteins and state transitions synergistically cooperate to induce nonphotochemical quenching during fluctuating light.

**Abbreviations**
CCM: CO_2_ concentration mechanism
C_i_
: inorganic carbon (CO_2_, HCO_3_^‐^, CO_3_^2‐^)
HL: high light
LHC: light‐harvesting complex
LHCSR: light‐harvesting stress related protein
NPQ: nonphotochemical quenching
PAM: pulse‐amplitude modulation
PS: photosystem
qE: energy‐dependent quenching
qI: photoinhibition
qT: state transitions
qZ: zeaxanthin‐dependent quenching
STT7: serine/threonine‐protein kinase
TCSPC: time‐correlated single photon counting

## 1 INTRODUCTION

Most life on Earth is sustained by photosynthetic organisms that capture sunlight energy to convert CO_2_ and water into chemical energy. Light is captured by light‐harvesting antenna complexes that contain a network of pigments absorbing photons and funneling the energy towards photosystems II and I that use it to perform photochemical reactions. Under light‐limiting conditions, efficient light harvesting is crucial for maximizing the rate of CO_2_ fixation (Björkman & Demmig, 1987). However, high light (HL) intensities can saturate reaction centers and lead to the build‐up of excess excitation energy, which, if unchecked, can lead to the production of reactive oxygen species and damage to both reaction centers (Khorobrykh et al., 2020). In nature, light exposure rapidly fluctuates in intensity with periods of HL ranging from milliseconds to hours (Graham et al., 2017), requiring photosynthesis to acclimate to different timescales of HL exposure. For each period of HL acclimation, photosynthetic organisms exhibit photoprotective mechanisms that regulate light harvesting and safely remove excess energy (Erickson et al., 2015; Pinnola & Bassi, 2018; Roach, 2020).

Upon light absorption, the energy can be dissipated as heat in a process called nonphotochemical quenching (NPQ). NPQ involves five components, each of which has been distinguished by its time of induction and relaxation during transition between dark and HL (Erickson et al., 2015). The fastest component, called energy‐dependent quenching (qE), is triggered by luminal acidification (Briantais et al., 1979) and is induced and relaxed within seconds. State transition (qT) occurs within minutes and involves the phosphorylation of light‐harvesting complexes (LHCs) (Allen, 1992) resulting in their detachment from Photosystem (PS) II and subsequent aggregation in a quenched state and/or association to PSI (Nagy et al., 2014; Nawrocki et al., 2016; Ünlü et al., 2014). Zeaxanthin‐dependent quenching (qZ) requires the accumulation of zeaxanthin and probably involves quenching in the minor LHCs of PSII (Dall'Osto et al., 2005; Nilkens et al., 2010; Wehner et al., 2006). On longer time scales, two more sustained forms of NPQ occur: qH that takes places in the antennae of PSII (Malnoë et al., 2018) directly in the LHCII trimers (Bru et al., 2021) and photoinhibition (qI) that occurs when degradation of the PSII core protein D1 exceeds its capacity for repair due to excess excitation energy (Aro et al., 1993).

In the green microalga *Chlamydomonas reinhardtii*, qE is mediated by pigment‐binding LHC stress‐related (LHCSR) proteins (Peers et al., 2009; Rochaix & Bassi, 2019). LHCSRs contain protonatable residues, which sense the decreasing luminal pH generated under HL conditions (Ballottari et al., 2016; Tian et al., 2019); the protonation of LHCSRs triggers NPQ within the protein (Kondo et al., 2017; Liguori et al., 2013; Troiano et al., 2021), allowing fast activation of qE. While there are two types of LHCSR proteins (LHCSR1 and LHCSR3), both of which bind pigments (Bonente et al., 2011; Perozeni et al., 2020), LHCSR3 (for which two homologs are present in *Chlamydomonas*) is thought to be the main actor in qE (Peers et al., 2009; Truong, 2011). On the other hand, qT is activated by the buildup of reducing equivalents in the thylakoid membrane, which activates a serine/threonine‐protein kinase (STT7) (Depege et al., 2003; Lemeille et al., 2009) that phosphorylates LHCII, enabling it to detach from PSII and ultimately reattach to PSI (Iwai, Takizawa, et al., 2010; Minagawa, 2011). While qZ has been described in *Chlamydomonas* (Niyogi et al., 1997), it does not seem to play a significant role in overall NPQ (Girolomoni et al., 2019; Tian et al., 2019), and its potential mechanism of action remains to be determined. Finally, while qH has not been described in *Chlamydomonas*, qI occurs upon continued excess illumination (Aro et al., 1993; Erickson et al., 2015) at the level of the PSII center, where oxygen‐mediated sensitization creates the irreversible formation of a quenching site (Nawrocki et al., 2021).

The photophysical and biochemical bases for NPQ have been studied for decades (Erickson et al., 2015), however, the in vivo operation has mostly been assessed under a single dark‐to‐HL transition (Nedbal & Lazár, 2021) leaving our understanding of photoprotection under more complex light patterns limited. While LHCSR and STT7 activity are both known to be important for steady‐state NPQ based on measurements performed under nonrepeating HL/dark periods (Allorent et al., 2013), their relative contributions to NPQ have not been quantified, and their response to faster‐timescale fluctuating light remains unstudied. Recent work has started looking at the response of NPQ to some specific light fluctuations in *Chlamydomonas* (Nawrocki et al., 2020; Roach, 2020) and in the moss *Physcomitrella* (Gao et al., 2022). However, the physiological role and the functioning of the NPQ components under the wide diversity of light patterns that are present in the natural environment is unexplored.

**Figure 1:**
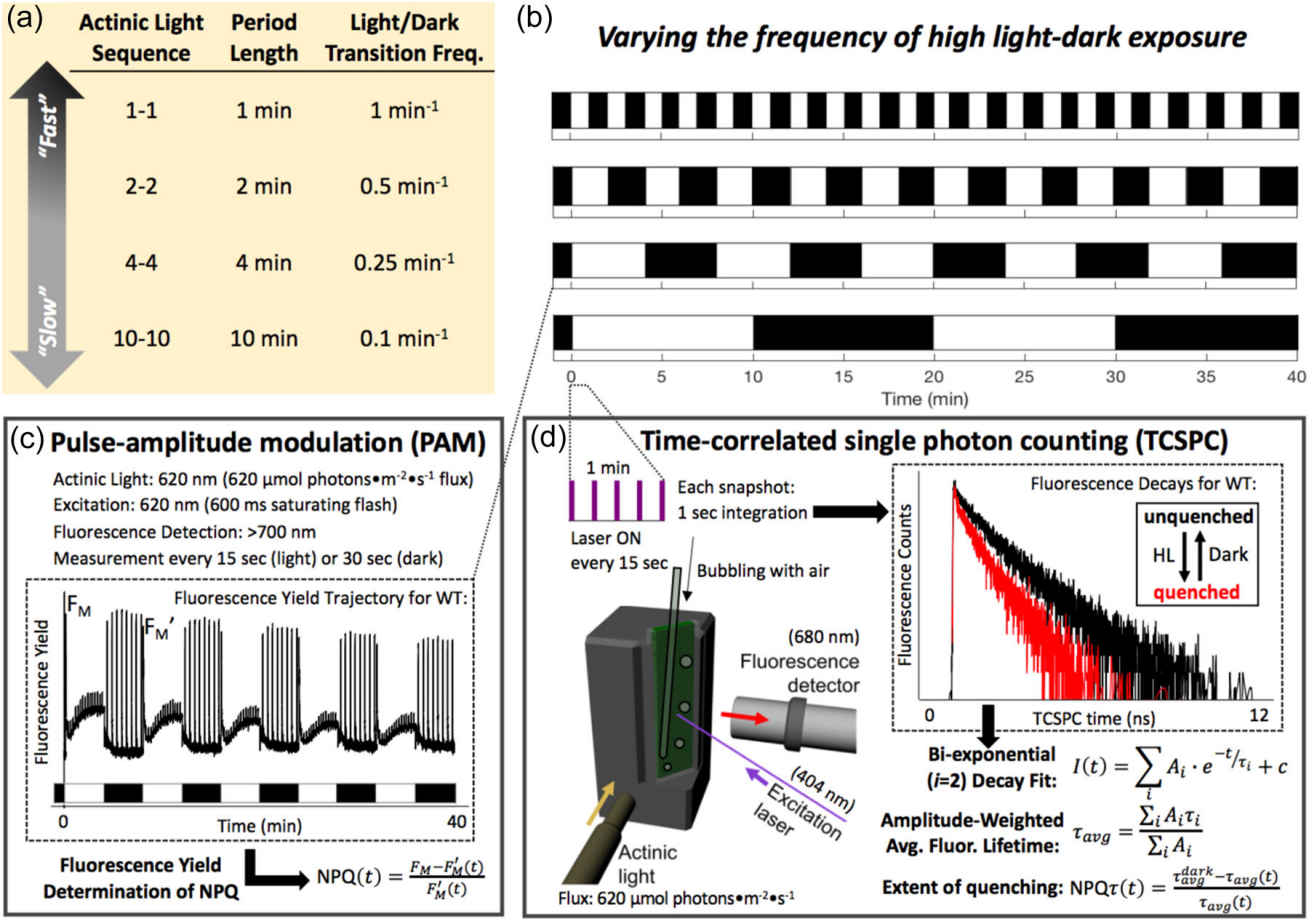
Experimental design for Chl *a* fluorescence measurements throughout exposure of *Chlamydomonas* cells to fluctuating light with various periods of high light (HL)‐dark exposure. (a, b) Representation of the HL‐dark cycles used for the 40 min of light fluctuation and their corresponding period, frequency, and name used throughout the main text. Nonphotochemical quenching (NPQ) was measured using pulsed‐amplitude modulation (PAM, c) and time‐correlated single‐photon counting (TCSPC, d). (c) Characteristics of the PAM measurement and representative data of fluorescence yield in WT cells. (d) Characteristics of the TCSPC apparatus. Shown are two decays representative of two snapshots taken in a quenched and unquenched state. WT, wild type [Color figure can be viewed at wileyonlinelibrary.com]

Here we utilized two distinct methods to monitor chlorophyll fluorescence in intact cells of *Chlamydomonas* that were exposed to various periodically fluctuating light sequences with HL/dark periods ranging from 1 to 10 min (Figure 1). The roles of qE and qT were investigated using single or double mutants of LHCSRs and STT7. Our analysis of LHCSR mutants (*npq4* [Peers et al., 2009], *lhcsr1* [Truong, 2011], and *npq4lhcsr1* [Truong, 2011]) revealed that LHCSR3 is the main contributor to the NPQ response during the HL phase of light fluctuations. Using mutants impaired in state transition (*stt7‐9* [Depege et al., 2003] and *stt7npq4* [Allorent et al., 2013]), we showed that qT quenching occurs primarily during the dark portion of the fluctuating light sequence and represents a significant part of NPQ during repeated light fluctuations. Our results showed that while qE and qT sustain most of the NPQ throughout light fluctuations, their relative importance varies during different phases of the fluctuating light response, with qT playing a larger role during dark periods and after repeated HL‐dark fluctuations. Surprisingly, the timescale of light fluctuations did not seem to have a major impact on the respective contributions of qE and qT although the contribution of qE during the dark phase was period‐dependent. Nonetheless, the various components of NPQ are not completely independent, and we reveal an interplay between LHCSR‐ and STT7‐mediated NPQ that enables the wild‐type photoprotective response. We further show that while *stt7* mutants are not impaired in growth under light fluctuations, short time scale light fluctuations highly impair LHCSR mutants. These findings represent an important first step in investigating the photosynthetic response to the diversity of HL periods that occur in nature.

## 2 MATERIALS AND METHODS

### 2.1 Strains and culture conditions

*Chlamydomonas* mutants and their respective wild‐type 4A‐ were previously described (*npq4* [Peers et al., 2009], *lhcsr1* [Truong, 2011], *npq4lhcsr1* [Truong, 2011], *stt7‐9* [Cardol et al., 2009], *stt7npq4* [Allorent et al., 2013]). Note here that because the *stt7‐9* mutant was obtained by complementing an arginine‐deficient strain (Depege et al., 2003) with a subsequent selection of a clone more prone to crossing (Cardol et al., 2009), it does not have a proper control strain. Accordingly, we used 4A‐ as the best possible control strain for all mutants. All strains were grown photoautotrophically under moderate light (50 µmol photons m^−2^ s^−1^) in minimal HS medium under air level of CO_2_ (20°C). Except for the growth test, cell cultures (5–8 µg Chl mL^−1^) were incubated overnight at high light (400 µmol photons m^−2^ s^−1^), for maximizing the expression of LHCSR proteins (Tibiletti et al., 2016). Before each measurement, cells were illuminated for at least 15 min with low intensity far‐red light (3in1LED panel with far‐red LED; 3LH series, NK system) to ensure a complete State 1 configuration (Bonaventura & Myers, 1969). All replicates shown are biological replicates from independent cultures.

### 2.2 Chlorophyll fluorescence measurements

In this study, we employ two techniques to monitor the activation and deactivation of NPQ throughout 40 min of exposure to repeated periods of high light and dark on the basis of changes in Chl fluorescence emission (see Figure 1 for an illustration of the experimental design). Time‐resolved Chl fluorescence was measured via time‐correlated single photon counting (TCSPC) while Chl fluorescence yield was measured in parallel experiments using pulse‐amplitude modulation (PAM) fluorimetry. Although both methods can monitor NPQ, the fluorescence lifetime is not susceptible to a range of nonquenching processes that can impact the fluorescence yield (such as chromophore bleaching, changes in chlorophyll concentration, chloroplast movement or enhanced light scattering [Sylak‐Glassman et al., 2016; Zaks et al., 2013]). Therefore, fluorescence lifetime measurements provide insight into processes that directly quench chlorophyll fluorescence.

#### 2.2.1 TCSPC measurement and fitting

The average chlorophyll fluorescence lifetime was measured by time‐correlated single‐photon counting (TCSPC), as previously described (Steen et al., 2020; Sylak‐Glassman et al., 2016). A diode laser (Coherent Verdi G10, 532 nm) pumped a Ti:Sapphire oscillator (Coherent Mira 900 f, 808 nm, 76 MHz) and the output was subsequently frequency doubled using a β‐barium borate crystal to obtain 404 nm light. These pulses were used for excitation of the sample with a power of 1.7 mW (20 pJ/pulse) and Chl fluorescence emission at 680 nm was detected via an MCP‐PMT (Hamamatsu R3809U). A custom‐built LABVIEW software was used to synchronize three shutters located in the laser path, actinic light path and the path between the sample and detector. Every 15s, a fluorescence lifetime snapshot measurement was acquired by exposing the cells to the saturating laser (404 nm) for 1 s and detecting the emission. Fluorescence lifetime snapshots were measured by TCSPC using a Becker‐Hickl SPC‐850 data acquisition card and SPCM software. In between the snapshot measurements, high‐light illumination of the cells was achieved by exposing the cuvette to white light set to an intensity of 620 μmol photons m^−2^ s^−1^ (Leica KL1500 LCD, peak 648 nm, FWHM 220 nm), chosen as the minimal light level necessary to saturate photosynthetic reactions and induce qE activity at the onset of illumination. It was also chosen such that PAM and TCSPC would use identical high light intensities. The sample concentration was adjusted to ~80 µg Chl ml^−1^ for TCSPC measurements. To control the gas composition of the culture and prevent cells from settling to the bottom of the cuvette, the sample was bubbled with air (ambient CO_2_ concentrations) at a rate of 2–7 ml min^−1^ throughout the entire 40 min experiment duration, although note that such bubbling increased the noise of the measurements.

For each fluorescence decay measurement, to ensure that PSII reaction centers were closed, we selected the 0.2 s step with the longest lifetime from the overall 1 s snapshot measurement duration (Sylak‐Glassman et al., 2016). This longest step was then fit to a bi‐exponential decay (Picoquant, Fluofit Pro‐4.6) and the average amplitude‐weighted fluorescence lifetime (τ_avg_) was calculated for each snapshot measurement. The NPQτ parameter is derived from the fluorescence lifetime snapshot measurements and is defined analogously to NPQ (Sylak‐Glassman et al., 2014, 2016): $\text{NPQ}\tau (t)=\frac{\tau_{\mathrm{avg}}(0)-\tau_{\mathrm{avg}}(t)}{\tau_{\mathrm{avg}}(t)}$. The value of NPQτ represents the magnitude of the quenching response based on the change in the average fluorescence lifetimes between time *t*=0 (after far‐red acclimation but before HL exposure) and any other time *t* during the 40 min snapshot trajectory. Therefore, using NPQτ removes confounding effects arising from any differences in the average chlorophyll excited state lifetime of the different strains following far‐red acclimation. For all TCSPC measurements, each biological replicate represents the average of three technical replicates measured on the same day.

#### 2.2.2 PAM measurements

Chlorophyll fluorescence yield was measured using a PAM fluorimeter (Dual‐PAM 100, Walz GmbH) with the red measuring head. Red saturating flashes (8000 µmol photons m^−2^ s^−1^, 600 ms, 620 nm) were delivered to measure *F*_M_ (maximal fluorescence yield in the dark‐acclimated samples) and then every 15 or 30 s to measure *F*_M_′ under actinic light exposure or dark phase, respectively. Actinic light illumination (620 nm) was set to 620 μmol photons m^−2^ s^−1^. Fluorescence emission was detected using a long‐pass filter (>700 nm). NPQ was calculated as (*F*_M_ − *F*_M_′)/*F*_M_′. The Chl concentration was ~5–8 µg Chl ml^−1^ and as for TCSPC, all PAM measurements and the sample was bubbled with air at a flux of 2–7 ml min^−1^ for proper control of the gas concentrations of the sample throughout the entire 40 min experiment duration, note that such bubbling increased the noise of the measurements (but to a lesser extent than for TCSPC).

#### 2.2.3 Quantifying the contributions of LHCSRs and STT7 to NPQ

To assess the relative contributions of LHCSR1, LHCSR3, and STT7 to overall NPQ, we analyzed the NPQ (PAM) and NPQτ (TCSPC) trajectories for WT and each mutant. The relative contribution of each protein was determined as the percent change in the integrated snapshot trajectory of NPQ or NPQτ for each mutant relative to the control WT strain. As the contribution of each actor was found to be overall independent of HL‐dark fluctuation frequencies in the range of 1–0.1 min^−1^. (Supporting Information: Figure 5), the average contribution of each protein under the four light fluctuating sequences for both PAM and TCSPC was considered. Additionally, to characterize the involvement in activation or deactivation of NPQ, the quenching trajectories were integrated solely under HL or dark periods, respectively (Supporting Information: Figure 6). The contributions of each protein to the early versus late responses were further assessed by integrating from 0 to 20 min and 20–40 min, respectively (Supporting Information: Figure 7). These results are summarized in Figure 6 of the main text. Since it has been suggested that the *stt7‐9* mutant may be “leaky” (Bergner et al., 2015), our estimates for the STT7 contribution are lower bound estimates. The potential leakiness of the *stt7‐9* mutant does not affect any of the conclusions presented in the manuscript. In addition to quantifying the relative contribution to NPQ of each protein relative to the WT strain, we also assessed the contribution of STT7 in the absence of LHCSR3 by integrating the PAM NPQ trajectory of *npq4stt7* relative to that of *npq4*. Likewise, the contribution of LHCSR3 was assessed independently of STT7 by integrating the PAM NPQ trajectory of *npq4stt7* relative to that of *stt7*.

### 2.3 77 K chlorophyll fluorescence emission

Chlorophyll fluorescence emission spectra of *Chlamydomonas* cells at 77 K were obtained by freezing whole cells (~5–8 µg Chl ml^−1^ final concentration) in liquid nitrogen. The emission spectrum was then measured between 600 and 800 nm (435 nm excitation wavelength, RF‐5300PC spectrophotometer, Shimadzu).

### 2.4 Growth tests

The different *Chlamydomonas* strains were cultivated photoautotrophically under moderate light (50 µmol photons m^−2^ s^−1^) in minimal medium under air level of CO_2_ (20°C). Cells were harvested during exponential growth and resuspended in fresh minimal medium to 0.1, 0.5, or 2 µg Chl ml^−1^. Eight‐microliter drops were spotted on minimal medium plates at pH=7.2 and exposed to the various light conditions for 8 days. Homogeneous light was supplied by LED panels. Temperature was maintained at 25°C at the level of plates.

### 2.5 Accession numbers

Genes studied in this article can be found on https://phytozome.jgi.doe.gov/ under the loci Cre08.g365900.t1.2 (LHCSR1), Cre08.g367500.t1.1 (LHCSR3.1), Cre08.g367400.t1.1 (LHCSR3.2), Cre02.g120250.t1.1 (STT7).

## 3 RESULTS

### 3.1 Varying light fluctuation periods affect the dynamic NPQ response

**Figure 2:**
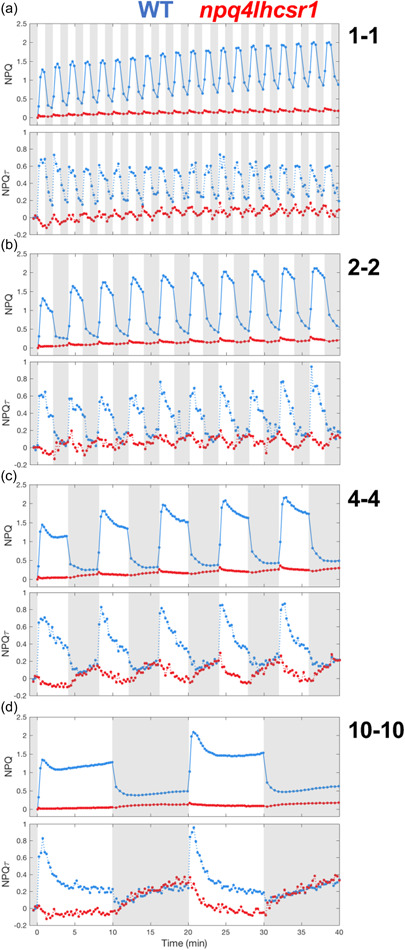
Quenching trajectories during light fluctuations in *npq4lhcsr1* and its control strain. The response of nonphotochemical quenching (NPQ) and NPQτ (upper and lower panel, respectively) were measured in *npq4lhcsr1* mutant and its control strain (red and blue curves, respectively) during 40 min of light fluctuations with periods of 1, 2, 4 and 10min (a, b, c and d, respectively) as described in Figure 1. Shown are the average of three biological replicates. For TCSPC data, each biological replicate was averaged from three technical replicates. The fluorescence lifetime values used to calculate NPQτ are shown in Supporting Information: Figure 13. [Color figure can be viewed at wileyonlinelibrary.com]

While the photosynthetic response of *Chlamydomonas* to some light fluctuations has been reported (Cantrell & Peers, 2017; Roach, 2020), an analysis of NPQ for various timescales of light fluctuations is lacking. We therefore measured chlorophyll fluorescence during periodic light‐dark cycles of 40 min with individual HL or dark periods ranging from 1 to 10 min (Figure 1). Chlorophyll fluorescence yield was measured using pulse‐amplitude modulated (PAM) fluorometry and used to calculate NPQ (Klughammer & Schreiber, 2008). In tandem experiments, time‐correlated single‐photon counting (TCSPC) was used to measure the chlorophyll fluorescence lifetime (Amarnath et al., 2012), which was used to calculate NPQτ (Sylak‐Glassman et al., 2014). For all timescales of light fluctuations in the wild‐type strain, NPQ quickly turned on upon illumination but turned off more slowly (Figure 2). The same trend was observed in NPQτ (Figure 2). The 1 min HL period light fluctuation led to a nearly square‐like response of NPQ and NPQτ (Figure 2).

For the longer HL periods ranging from 2 to 10 min, after an initial burst of NPQ in HL, the level of NPQ decreased with continued illumination, eventually reaching a steady state for the 10 min HL period (Figure 2). A similar trend was also seen in NPQτ (Figure 2). Therefore, these kinetics are directly related to chlorophyll fluorescence quenching, rather than other nonquenching processes that affect chlorophyll fluorescence. This phenomenon of decreasing NPQ during the light has been previously attributed to the consumption of the proton gradient by the activity of the CO_2_ concentration mechanism (CCM) (Burlacot et al., 2022). In addition to the effect arising from CCM, state transition via the induction of State 1 could also play some role in the NPQ decrease observed during HL periods, though this would not contribute during the first HL period in which cells are already in State 1 (following far‐red acclimation). For fluctuating light periods longer than 4 min, upon a transition from HL to dark, the NPQ turned off rapidly but was then followed by a gradual rise in NPQ during further darkness, a trend that was also observed in NPQτ (Figure 2). However, compared with NPQ, NPQτ showed a larger magnitude of increase during the long dark periods (Figure 2c,d). Differences between the NPQ and NPQτ traces are considered in the discussion.

We conclude from these experiments that, when exposed to periodic light fluctuations with HL periods ranging from 1 to 10 min, at least three components of NPQ are present: (i) a rapidly responding component, (ii) a slowly inducible component induced throughout the light fluctuations and (iii) a component induced in the dark phases of light fluctuations.

### 3.2 The majority of NPQ during light fluctuations is mediated by LHCSR proteins

**Figure 3:**
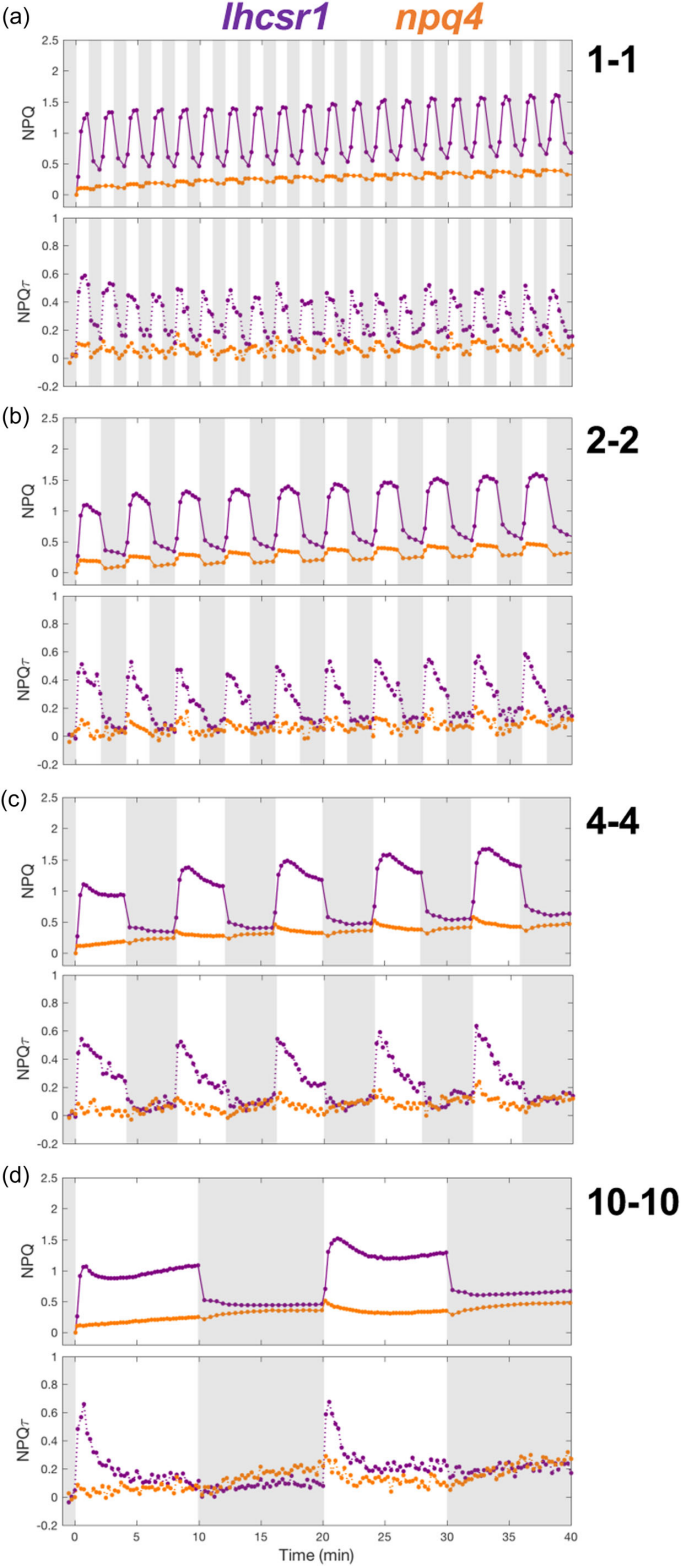
Quenching trajectories during light fluctuations in *lhcsr1* and *npq4*. The response of nonphotochemical quenching (NPQ) and NPQτ (upper and lower panel, respectively) were measured in *lhcsr1* and *npq4* (purple and orange curves, respectively) during 40 min of light fluctuations with periods of 1, 2, 4 and 10min (a, b, c and d, respectively) as described in Figure 1. Shown are average of three biological replicates. For TCSPC data, each biological replicate was averaged from three technical replicates. The fluorescence lifetime values used to calculate NPQτ are shown in Supporting Information: Figure 14. [Color figure can be viewed at wileyonlinelibrary.com]

It has been well established that LHCSR proteins are crucial for NPQ in *Chlamydomonas* during a single dark‐to‐light transition (Correa‐Galvis et al., 2016; Peers et al., 2009; Truong, 2011). To examine the relative importance of each LHCSR protein for the functioning of NPQ during light fluctuations, we measured the chlorophyll fluorescence yield and lifetime during the same light‐dark cycles on mutants impaired in the accumulation of LHCSR1 (*lhcsr1*) (Truong, 2011), LHCSR3‐1 and LHCSR3‐2 (*npq4*) (Peers et al., 2009), or all three LHCSRs (*npq4lhscr1*) (Ballottari et al., 2016; Truong, 2011). While the *npq4lhcsr1* mutant was highly impaired in its NPQ capacity for all light fluctuations (Figure 2), single *npq4* and *lhcsr1* mutants showed some NPQ in response to light fluctuations (Figure 3). Noticeably, for HL periods longer than 4 min, the increasing NPQ observed during dark phases was more pronounced in the *npq4lhcsr1* mutant (Figure 2). We conclude from these experiments that although LHCSRs are responsible for most of the NPQ during the light phase of all light fluctuations, a substantial portion of the NPQ in WT is nonetheless mediated by other biochemical processes, part of which is induced during the dark periods of light fluctuations.

### 3.3 The increasing quenching in the dark periods arises from state transition

**Figure 4:**
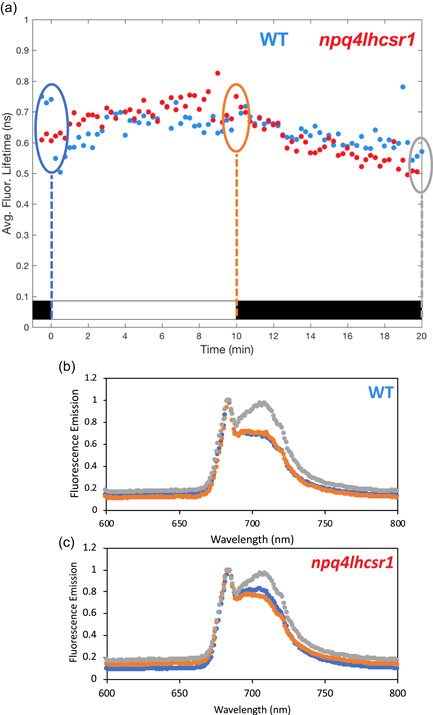
77 K chlorophyll fluorescence emission spectra during the first high light‐dark cycle of light fluctuations. Cells were placed in a time‐correlated single photon counting (TCSPC) cuvette as described in Figure 1 and both fluorescence lifetime snapshots and 77K chlorophyll fluorescence emission spectra were taken through 10 min of high light and 10 min darkness. (a) Fluorescence lifetime trajectory of *npq4lhcsr1* mutant (red dots) and its control strain (WT, blue dots). On the graph, dashed vertical lines depict the timepoints at which samples were taken for 77 K fluorescence spectra measurement. (b, c) 77 K fluorescence emission spectra of samples taken in (a) on the control strain (WT, b) and *npq4lhcsr1* mutant (c). Spectra were taken at 0, 10 and 20 min timepoints (blue, orange and grey spectra, respectively). Shown are representative spectra. Three independent biological replicate spectra for WT and *npq4lhcsr1* are shown in Supporting Information: Figure 1. 77 K spectra for the *s tt7* and *stt7npq4* strains are shown in Supporting Information: Figure 2. [Color figure can be viewed at wileyonlinelibrary.com]

**Figure 5:**
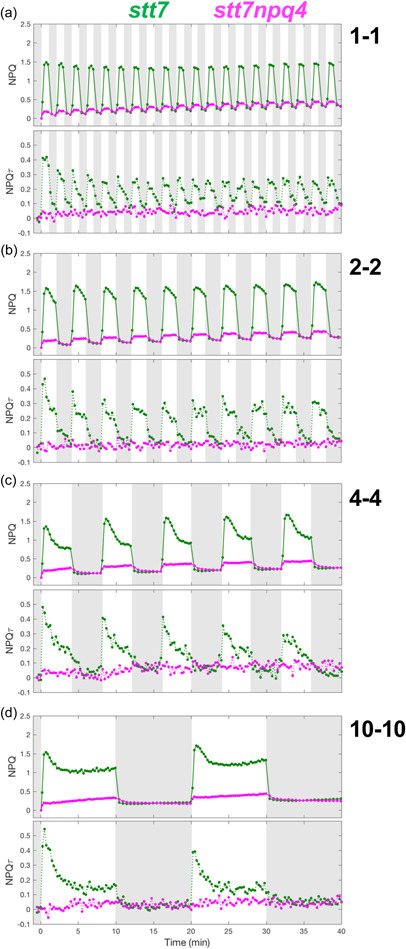
Quenching trajectories during light fluctuations in *stt7* and *stt7npq4*. The response of nonphotochemical quenching (NPQ) and NPQτ (upper and lower panel, respectively) were measured in *stt7* and *stt7npq4* (green and magenta curves, respectively) during 40 min of light fluctuations with periods of 1, 2, 4 and 10 min (a, b, c and d, respectively) as described in Figure 1. Shown are the average of three biological replicates. For TCSPC data, each biological replicate was averaged from three technical replicates. The fluorescence lifetime values used to calculate NPQτ are shown in the Supporting Information: Figure 15. [Color figure can be viewed at wileyonlinelibrary.com]

Induction of NPQ during darkness has been previously reported in chlorophytes (Allorent et al., 2013; Casper‐Lindley & Björkman, 1996), and qT has been proposed to be involved (Allorent et al., 2013). Reorganization of light‐harvesting antennae between PSII and PSI was thus followed throughout a light fluctuation by measuring 77K fluorescence emission spectra for cells at three time points: after acclimation to far‐red light (cells in State 1 [Zhang et al., 2021]), after 10 min HL, and after 10 additional min dark (see vertical lines in Figure 4a). In WT and *npq4lhcsr1*, an increase in the emission at 710 nm specific to PSI‐bound LHCII was observed between the 10 (after HL) and 20 min (after dark) time points, suggesting that some reassociation of LHCII from PSII to PSI occurs during the dark portion of our measurements (Figure 4, Supporting Information: Figure 1). In contrast, mutants lacking the STT7 kinase responsible for qT (*stt7* and *stt7npq4*) showed negligible changes in the 77 K fluorescence emission spectra (Supporting Information: Figure 2) and only showed a minimal increase in NPQ or NPQτ during the dark periods of light fluctuations (Figure 5) and with increasing duration of exposure to light fluctuations (Supporting Information: Figure 3).

To characterize the kinetics of qT occurring during the light‐to‐dark transition, we analyzed the response of the chlorophyll fluorescence lifetime for WT and *npq4lhcsr1* mutant cells that were exposed to 10 min of HL followed by 30 min of dark. Interestingly, upon light‐to‐dark transition, both strains showed steadily decreasing lifetimes for the first 10 min of darkness, after which, the fluorescence lifetimes began to reverse, eventually reaching the starting lifetime after 30 min of darkness (Supporting Information: Figure 4). In contrast, *stt7* showed no such quenching of fluorescence lifetime during the 10 min dark period. Therefore, we conclude that the quenching observed during the dark phases of light fluctuations in *Chlamydomonas* arises from qT, which has an induction timescale of ~10 min and is reversible upon continued exposure to darkness.

### 3.4 Contributions of qE and qT to NPQ and growth under fluctuating light

**Figure 6:**
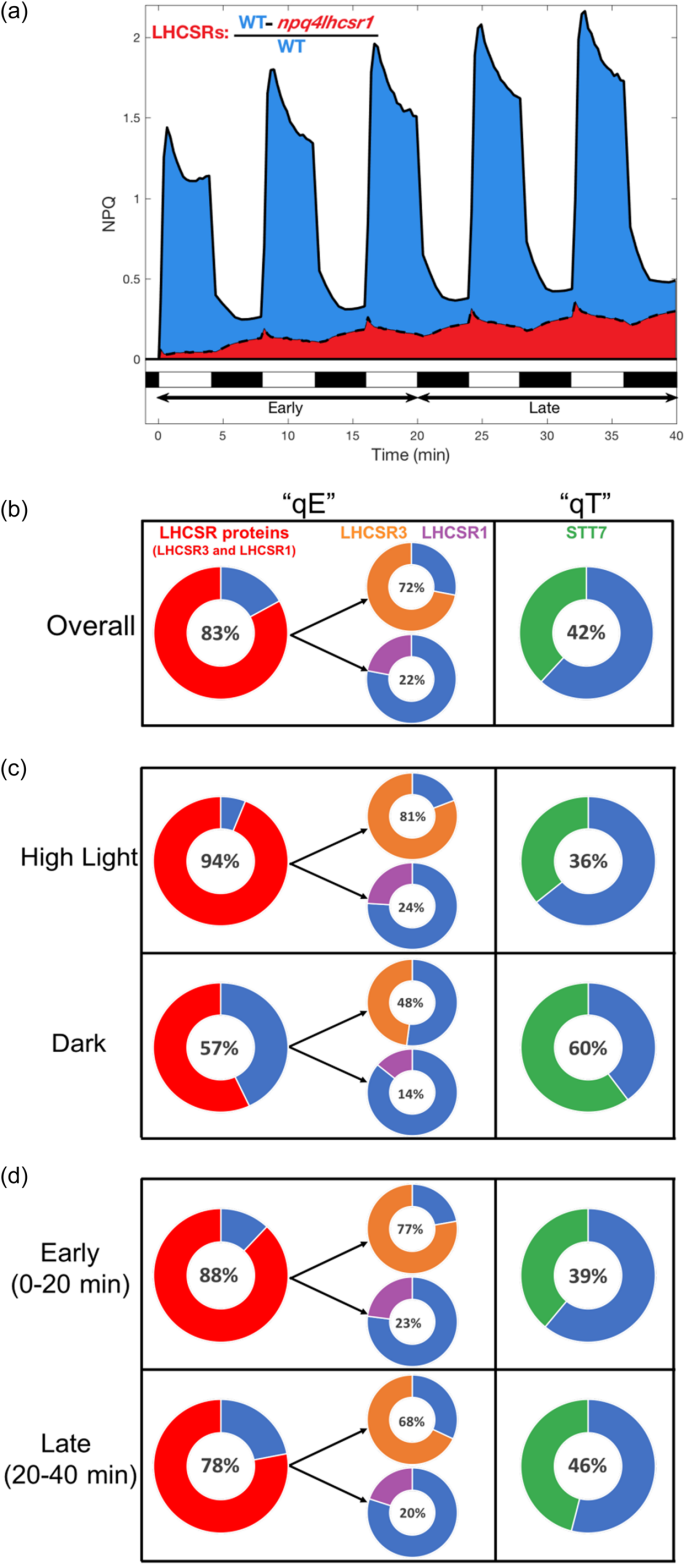
Quantification of the contribution of light‐harvesting complex stress‐related (LHCSRs) and STT7 to wild‐type nonphotochemical quenching (NPQ) under fluctuating light. (a) Example of quantification of the relative NPQ mediated by LHCSRs. The area under the NPQ curve of *npq4lhcsr1* mutant (red) was subtracted from that of the control strain (blue) and expressed relative to the area of NPQ of the control strain. (b, c) Overall contribution of LHCSRs (red), LHCSR3 (orange), LHCSR1 (purple) and STT7 (green) averaged over all 40 min (b) or specific periods of the light fluctuations (c). For each protein, the part of the donut shown in red, orange, purple or green is the percentage of wild‐type NPQ lost in each mutant impaired in the accumulation of LHCSRs, LHCSR3, LHCSR1 and STT7, respectively. Given that the contribution of each protein was largely independent of HL/dark period (Supporting Information: Figure 6), shown are the average of all four light fluctuation sequences. Distribution of individual replicates and estimates of error (i) overall, (ii) during HL or dark and (iii) during early and late portion of light fluctuations are presented in Supporting Information: Figures 5, 6, and 7, respectively. [Color figure can be viewed at wileyonlinelibrary.com]

**Figure 7:**
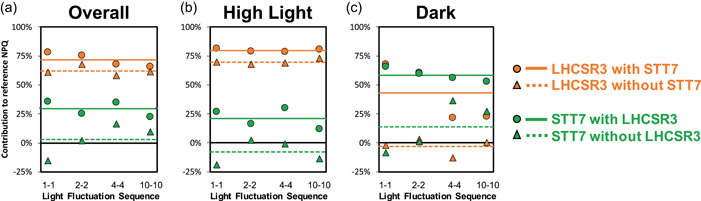
Untangling qE and qT. The quantification of the contributions of LHCSR3 (orange) and STT7 (green) to the nonphotochemical quenching (NPQ) response both in the presence (solid lines, circle markers) and absence (dashed lines, triangle markers) of the other protein is plotted as a function of the light fluctuation period. Horizonal lines show the average contribution for each protein, considering all light fluctuation frequencies. For reference, the black lines represent zero contribution. The contribution of each protein was evaluated (a) over all 40min of the experiment, (b) only during HL periods, or (c) only during dark periods. All of these were evaluated from PAM trajectories using various combinations of WT along with the *npq4*, *stt7* and *stt7npq4* mutants as discussed in Section 2. [Color figure can be viewed at wileyonlinelibrary.com]

It has been previously proposed that, while LHCSRs play an important role during short timescale illumination, state transitions are important for the cell's longer timescale response to HL acclimation (Erickson et al., 2015). To test this hypothesis and assess the scenario under fluctuating light conditions, we quantified the amount of NPQ that was mediated by each protein by comparing the remaining NPQ (or NPQτ) in each mutant relative to the NPQ (or NPQτ) in the WT reference strain (Figure 6a, Table 1). Surprisingly, the contribution of each protein to overall NPQ did not seem to depend on the period of the light fluctuation (Supporting Information: Figure 5). Therefore, as a first estimate for the contribution of LHCSRs and STT7, we calculated the average contribution under all four light fluctuation sequences. The donut charts in Figure 6b,c,d depict the contribution of each protein as a percentage of the total NPQ observed in the WT strain (represented as the complete donut). While LHCSR3 is responsible for the majority of overall NPQ (72%, Figure 6, 7), STT7 had a substantial contribution mediating 42% of the NPQ, with LHCSR1 having a smaller contribution at 22% of NPQ. LHCSRs were found to have a substantially larger contribution during light phases, where they are responsible for 94% of WT NPQ, while in the dark phases their contribution declined to 57% (Figure 6c, Supporting Information: Figure 6). On the other hand, STT7 played a significantly larger role in the NPQ during darkness (60%) than it does during illumination (36%) this latter result being likely due to inhibition of STT7 by high light (Allorent et al., 2013; Rintamäki et al., 2000; Vink et al., 2004). Interestingly, the amount of NPQ mediated by LHCSRs was more important during the beginning of light fluctuations while state transitions contributed more after 20 min of light fluctuation (Figure 6d, Supporting Information: Figure 7), revealing increased relative contribution of qT and decreasing contribution of qE with increasing time of exposure to light fluctuations.

**Table 1:** Average contribution of each protein to overall wild‐type NPQ for each light fluctuation sequence

Overall contributions	1‐1	2‐2	4‐4	10‐10	**Average**
LHCSRs	89 ± 7%	84 ± 7%	83 ± 13%	77 ± 24%	83 ± 5%
LHCSR1	22 ± 12%	22 ± 10%	24 ± 16%	19 ± 30%	22 ± 2%
LHCSR3	81 ± 5%	78 ± 5%	73 ± 8%	58 ± 15%	72 ± 10%
STT7	46 ± 12%	38 ± 16%	46 ± 13%	39 ± 22%	42 ± 4%

*Note*: Shown is the average value (*n *= 6, evaluated from three TCSPC and three PAM replicates) and standard deviation of all individual replicates. The contributions of LHCSR3 (orange) and LHCSR1 (purple) were determined from the single mutants npq4 and lhcsr1. The contribution of LHCSRs overall (red) was evaluated from the npq4lhcsr1 mutant. The contribution of qT was assessed from the stt7 mutant. Each error in the right column represents the standard deviation of each protein's contribution across the four light fluctuation sequences. For simplicity, only the average values (shown in the right column) were used to generate Figure 6 in the main text. Full distributions of the individual TCSPC and PAM data points are shown in Supporting Information: Figure 5.

Since LHCSR3 is part of the mobile fraction of photosynthetic antenna and has been proposed to be affected by STT7 activity (Allorent et al., 2013), giving a potential mechanistic connection between qE and qT (Roach & Na, 2017), we aimed to interrogate the involvement of qT both together and separately from qE under fluctuating light. We compared the integrated PAM NPQ trajectories of *npq4stt7* relative to that of *npq4*, both of which lack LHCSR3, or *npq4stt7* relative to that of *stt7*, both of which lack STT7. The relative contribution of STT7 to NPQ was found to be different in the presence versus absence of LHCSR3, with STT7 playing a larger role when LHCSR3 is present (Figure 7 and Table 2). Likewise, the contribution of LHCSR3 was larger when STT7 was present. These data suggest that STT7 and LHCSR3 synergistically cooperate to induce quenching during dark periods of light fluctuations. We propose that the activity of STT7 resulting in LHCSR phosphorylation and antenna mobility is responsible for this crosstalk between qE and qT during light fluctuations.

**Table 2:** Average contribution of LHCSR3 and STT7 in the presence and absence of the other protein for each light fluctuation sequence

Overall contributions	1‐1	2‐2	4‐4	10‐10	Average
LHCSR3 w/STT7	78 ± 5%	75 ± 5%	67 ± 6%	66 ± 14%	72 ± 6%
LHCSR3 w/out STT7	61 ± 5%	67 ± 4%	58 ± 3%	61 ± 3%	62 ± 4%
STT7 w/LHCSR3	36 ± 4%	25 ± 11%	35 ± 8%	22 ± 9%	29 ± 7%
STT7 w/out LHCSR3	−16 ± 15%	2 ± 12%	16 ± 5%	9 ± 6%	2 ± 14%

*Note*: Shown is the average value (evaluated from three PAM replicates) and standard deviation of individual replicates. Each error in the right column represents the standard deviation of the four light fluctuation sequences. For simplicity, only the average values (shown in the right column) were used to generate Figure 7a in the main text.

**Figure 8:**
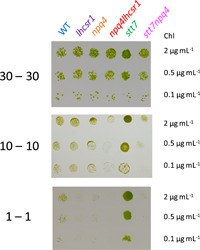
Growth of mutants impaired in qE and/or qT under various periods of dark/light cycles. *lhcsr1*, *npq4*, *npq4lhcsr1*, *stt7* and *stt7npq4* mutants and their control strain (WT) were diluted and spotted at different chlorophyll concentration and grown on plates under dark/light cycles with a period of 30 (30‐30, upper panel), 10 (10‐10, middle panel) or 1 min (1‐1, lower panel). Each row represents a different chlorophyll concentration. Shown are representative spots of three biological replicates. Cells were grown for eight days. Growth under constant low light or high light are shown in Supporting Information: Figure 8. [Color figure can be viewed at wileyonlinelibrary.com]

Although mutants impaired in the accumulation of LHCSRs and STT7 have strongly impaired NPQ capacities, this does not seem to impair the growth of those strains under continuous high light conditions (Cantrell & Peers, 2017; Depege et al., 2003; Peers et al., 2009). Recent data have shown that the growth of *npq4* and *npq4lhcsr1* mutants is impaired under certain light fluctuation conditions (Cantrell & Peers, 2017; Roach, 2020). We therefore investigated whether the growth impairment of those strains could be dependent on the timescale of light fluctuations. While all the mutants grew as well as the control strain under continuous illumination (Supporting Information: Figure 8), *npq4*, *lhcsr1*, *npq4lhcsr1* and *stt7npq4* mutants exhibited impaired growth under fast light fluctuations with a 1‐min HL period (Figure 8). In contrast, only *npq4lhcsr1* and *stt7npq4* mutants showed an impaired growth under slower light fluctuations with an HL period of 10 min, and the growth of all mutants was similar when the HL period was increased to 30 min (Figure 8). We conclude from this experiment that qE mediated by LHCSR proteins is critical for growth under light fluctuations and that this role is more important for rapid light fluctuations.

## 4 DISCUSSION

The involvement of pH‐sensing LHCSR proteins and state transitions in the photoprotective response of *Chlamydomonas* has been previously described (Allorent et al., 2013; Peers et al., 2009). While mutants impaired in accumulation of LHCSRs were shown to have limited growth when light is provided in a day/night cycle (Cantrell & Peers, 2017) or fluctuating with a 10‐min timescale (Roach, 2020), our understanding of the contribution of LHCSRs and state transitions to photoprotection during fluctuating light remains limited. Here, by measuring the NPQ levels during light fluctuations in a range of mutants impaired in the accumulation of LHCSR3, LHCSR1 and/or STT7 we have unraveled their relative contributions to NPQ. Varying the timescale of fluctuating light periods from 1 to 10 min allowed us to assess the dynamics of rapid qE‐ and slower qT‐type processes. Interestingly, we observe that qT builds up during the dark periods of the light fluctuations and continues to play a role in the NPQ response during subsequent light phases. It occurs even in the absence of LHCSR3 (see *npq4* and *npq4lhcsr1* mutant in Figures 2 and 3), has a timescale of 10 min, and is reversible (Supporting Information: Figure 4), which is consistent with recent literature (Allorent et al., 2013; Zhang et al., 2021). During a transition between low‐light and high‐light stress, qE proteins take a few hours to be fully induced (Peers et al., 2009), and it was hypothesized that qT may substitute for qE during this delay (Allorent et al., 2013). Our results show that even when LHCSRs are fully activated (i.e., in high light‐acclimated cells), the occurrence of qT remains substantial during light fluctuations (Figure 2). State transition or qE mutants were previously shown to have high reactive oxygen species (ROS) production (Allorent et al., 2013; Barera et al., 2021). Thus, the substantial amount of qT induced during the dark periods of light fluctuations may enhance photoprotection and limit ROS production by “anticipating” the next exposure to high light. The combination of fast qE (turns on rapidly upon HL exposure due to ΔpH) and residual qT (from previous dark periods) could therefore provide effective photoprotection in an unpredictable fluctuating‐light environment.

Since qE has long been ascribed as the fastest component of NPQ, directly responding to the thylakoid lumen proton concentration (Briantais et al., 1979), and qT as a slower component (Allorent et al., 2013), the contribution of qE to NPQ was proposed to be stronger for short periods of HL, with the contribution of qT becoming increasingly important during longer periods of high‐light stress (Erickson et al., 2015). Our approach of systematically assessing the response of photosynthesis to various timescales of light fluctuations has revealed nuances in this interpretation. Surprisingly, we found that the overall contributions of qE and qT are different in the presence versus absence of the other component (Figure 7) revealing that interactions between qE and qT contribute to the NPQ response during all timescales of light fluctuation. Additionally, the *npq4* mutant showed significantly reduced NPQ capacity compared with WT in the dark periods (by 74%) under fast light fluctuations (1‐1 sequence), but only a 17% impairment under slower fluctuations (10‐10) (see Supporting Information: Figure 6b), showing that relaxation of qE (around 1 min) is mediated by LHCSR3 and contributes substantially to the response of NPQ to rapid light fluctuations. Such relaxation kinetics may contribute to a faster response of NPQ to the next illumination if the period of light fluctuations is shorter than 2 min. It is also worth noting that the relative importance of qE in NPQ decreased after 20 min of light fluctuations, while the opposite occurred for qT (Figure 6), reflecting a build‐up in qT throughout the 40 min of light fluctuations. Modeling the response of photosynthesis to complex light fluctuations has been done (Tanaka et al., 2019; Zaks et al., 2012, 2013; Steen et al. 2020; Nedbal & Lazár,) and would allow targeting specific mechanisms for increasing plant yields in the field. Our results show that such efforts should consider both the period of light and dark as well as the total time exposed to fluctuating light. The light intensities used during light fluctuations may also affect the relative contributions of each mechanism. In green microalgae, it is tempting to speculate that in nature, where exposure to HL and dark occur repeatedly, qE may play a more important role in the beginning of light fluctuations while qT may provide a photoprotective response on a longer timescale.

Although qI is known to contribute to NPQ during prolonged illumination, it is likely negligible in our experimental conditions since both *npq4lhcsr1* and *npq4stt7* mutants did not show a strong buildup of NPQ or NPQτ during the 20 min of HL exposure during the light fluctuations (Figures 2 and 5). Additionally, Fv/Fm were not significantly different between strains before light fluctuations, with the exception of *npq4lhcsr1* which showed a lower Fv/Fm (Supporting Information: Figure 9). Likewise, the *npq4lhcsr1* double mutant showed a shorter starting fluorescence lifetime (Figure 4a), which likely reflects some constitutive qI following overnight HL acclimation in the complete absence of LHCSR proteins.

Interestingly, when comparing NPQ and NPQτ, the magnitude of the quenching decrease during HL was larger for NPQτ than NPQ (Figure 2). The energetic requirement (and thus its proton gradient consumption) of the CCM depends on the inorganic carbon (C_i_) availability (Fei et al., 2022). Since the high cell concentration in the TCSPC sample leads to strong C_i_ consumption, this could deplete the C_i_ concentration even in the presence of bubbling, leading to a C_i_ level sensed by cells in the TCSPC sample being lower than what is experienced by cells in the PAM sample. This would lead to higher activity of CCM, and hence a larger decrease in quenching, under TCSPC sample conditions compared with PAM sample conditions. The decrease in both NPQ and NPQτ was also stronger for longer HL periods as well as later in the fluctuating light sequence (Figure 2). The slope of the initial decrease in NPQτ or NPQ during HL was similar for all four sequences (Supporting Information: Figure 10) and is likely dictated by C_i_ availability and its influence on the CCM kinetics. The magnitude of the decrease in NPQ and NPQτ was larger for longer light periods (Supporting Information: Figure 10d,h), likely due to simultaneous activation of the CCM that dissipates the proton gradient and the onset of slower forms of NPQ such as state transitions. Conversely, the differences in the magnitude of the increase in NPQ and NPQτ during the dark periods could be due to differences in O_2_ concentrations sensed by the cells in both conditions, which is known to affect the extent and rate of state transition (Forti & Caldiroli, 2005).

Although LHCSR3 plays the dominant role in photoprotection under constant and fluctuating light conditions, we also observe a role for LHCSR1 in our measurements. While the chlorophyll fluorescence dynamics of *lhcsr1* are similar to those of WT (Figure 3), we observe a ~20% reduction in overall NPQ in this mutant under fluctuating light conditions (Figure 6). This small amount of photoprotection afforded by LHCSR1 in vivo is consistent with previous in vitro investigations (Dinc et al., 2016; Nawrocki et al., 2020) in which LHCSR1 has been suggested to mediate energy transfer between LHCII and PSI (Kosuge et al., 2018) and to compensate for the absence of LHCSR3 (Girolomoni et al., 2019). Interestingly, our results suggest that different from the case for LHCSR3, the qE that is mediated by LHCSR1 is largely independent of the timescale of light fluctuations (Supporting Information: Figure 5). Both LHCSR1 and LHCSR3 are thought to generate NPQ in response to (i) the proton gradient and (ii) carotenoid composition (Kondo et al., 2017; Troiano et al., 2021), thus the frequency‐dependent LHCSR3 and frequency‐independent LHCSR1 could differ in their dependence on the pH or carotenoid.

The relationship between NPQ capacities and growth have remained puzzling in *Chlamydomonas*, because only some light fluctuation regimes have consistently been shown to impair growth (Cantrell & Peers, 2017; Peers et al., 2009; Roach, 2020; Truong, 2011). Here we show that all mutants lacking LHCSRs showed impaired growth under rapid light fluctuations (1‐1 sequence), and that this impairment was lower under slower fluctuations (10‐10 sequence) and absent under an even slower 30‐30 sequence or constant illumination (Figure 8 and Supporting Information: Figure 8). There seems to be a good relationship between defect of NPQ and growth deficiency under rapid fluctuations when considering *npq4lhcsr1* and *stt7npq4* mutants (Figure 8), clearly showing that LHCSR‐dependent qE is critical for growth when light fluctuates within a short period of time. However, surprisingly, the growth defect of *lhcsr1* seemed larger than *npq4* under the 1‐1 sequence. This suggests that the growth capacity of LHCSR mutants under light fluctuations may not depend only on the level of NPQ. The growth defects observed for qE mutants under short timescale light fluctuations may be affected by the deactivation of the Calvin–Benson–Basham (CBB) cycle during the dark phases, as previously proposed by (Roach, 2020). Such a frequency dependence for the growth may thus additionally reveal the specific time scales of CBB cycle activation and deactivation during extended periods of fluctuating light. Other factors may include the activation of compensatory mechanisms that enable photoprotection at the expense of growth or the increased production of reactive oxygen species in *npq4* mutants (Barera et al., 2021; Roach et al., 2020), which could be greater in the *lhscr1* mutant. However, the NPQ dynamics may be different for cells grown on plates than in liquid medium due to differences in nutrient availability such as nitrogen or CO_2_. These differences would limit the validity of our conclusions regarding the growth outcomes under fluctuating light. Note here that in our conditions, due to slightly different genetic background between *stt7* and the WT control, we cannot make a conclusion on the mechanism by which *stt7* grew better under rapid timescale fluctuations (Figure 8). The WT background may be particularly sensitive to fast 1‐1 and 10‐10 fluctuations; another possibility could be that qT is detrimental for growth under medium to rapid timescale fluctuations.

Through 77 K fluorescence emission spectra analysis, we have shown that the increasing dissipation observed in the dark requires the STT7 kinase responsible for state transition **(**Figure 4 and Supporting Information: Figure 2). This effect, already described in *Chlamydomonas* (Allorent et al., 2013) and *Dunaliella salina* (Casper‐Lindley & Björkman, 1996), greatly contributes to NPQ during light fluctuations. In plants, the occurrence of state transitions and its involvement in NPQ is thought to be minor (Allen, 1992; Minagawa, 2011) even if mutants of *Arabidopsis thaliana* impaired in state transition (*stn7*) exhibit impaired growth under light fluctuations (Bellafiore et al., 2005). Interestingly, an increase of NPQ during the dark periods of fluctuating light was recently reported in *npq4* leaves of *Arabidopsis thaliana* (Steen et al., 2020) for which about 53% of the WT NPQτ remained in the mutant after 40 min of exposure to light fluctuations despite the absence of the pH‐sensing protein PsbS (Supporting Information: Figure 11). However, it remains unclear as to how much of the dark quenching in plants originates from qT as opposed to effects related to de‐epoxidized xanthophyll pigments (Steen et al., 2020) or LHC protein conformation and/or aggregation (Goral et al., 2012). In the future, periodic illumination experiments performed on *A. thaliana* mutants impaired in qE and/or qT could clarify the relative importance of each mechanism and allow a comparison of the in vivo functioning of NPQ in higher plants and green algae under light fluctuations.

Tuning the relaxation kinetics of NPQ in higher plants has been shown to improve crop plant productivity (Kromdijk et al., 2016), and recent modelling of the response of plant canopies to natural light fluctuations has shown that there remains ample room for improving photosynthetic efficiency under nonsteady‐state conditions (Wang et al., 2020). Similar opportunities for improvement also exist for increasing biofuel production from microalgae (Benedetti et al., 2018; Perin & Jones, 2019; Vecchi et al., 2020). In both cases, such optimization will require detailed understanding of the dynamic activity of NPQ mechanisms. The sum of the NPQ contributions of each protein is greater than 100% (Figure 6) which cannot be explained by a difference in the amount of LHCSR3 between WT and *stt7* (Supporting Information: Figure 12). Additionally, our quantifications of qE and qT reveal that each of them depends on the presence of the other process (Figure 7), showing that there is an interaction between qE and qT in *Chlamydomonas* under fluctuating light. The possibility of an interaction between qE and qT has been previously suggested on the basis of a kinetic analysis of qT in the presence and absence of LHCSR3 protein (*npq4* mutant) (Roach & Na, 2017). Our findings are consistent with a partial overlap of the functions of LHCSR3 and STT7 in both qE and qT. This partial overlap highlights the need for further investigations of the interactions occurring between proteins that underlie the in vivo NPQ response, not only in HL but also in the dark, and more generally during light fluctuations. For example, in microalgae, the quenching mediated by LHCSR3 both at PSII and PSI levels (Girolomoni et al., 2019) could be tuned by the movement of LHCSR3 from PSI to PSII during state transitions (Allorent et al., 2013). Thus, the described LHCSR3 phosphorylation by STT7 (Bergner et al., 2015) could explain the crosstalk between qE and qT. LHCSR3 is also known to associate with LHCII trimers in the PSII supercomplex (Semchonok et al., 2017); therefore, a similar LHCSR3–LHCII interaction may also generate quenching in the trimers following the detachment of LHCII from PSII. It should be noted that in the thylakoid membrane, LHCII can exist in a range of different conformations and/or quenching states (Kawakami et al., 2019; Tian et al., 2015). At this point, it is not possible to distinguish the relative contributions of different forms of LHCII in individual snapshot measurements. The ensemble fluorescence lifetime likely originates from some combination of at least three LHCII subpopulations: unphosphorylated and bound to PSII (State 1) (Drop et al., 2014) with an intermediate fluorescence decay component, phosphorylated and unbound (free LHCII) (Iwai, Yokono, et al., 2010) which has been previously assigned to a long fluorescence decay component (Ünlü et al., 2014), and phosphorylated and bound to PSI (State 2) (Huang et al., 2021) with a short fluorescence decay component. In intact algal cells, the relative abundance of each form of LHCII likely dynamically evolves throughout exposure to the fluctuating HL‐dark sequences. Further development of in vivo spectroscopic tools will be required to disentangle the dynamics of LHCII conformations and correlate them with photoprotection. Overall, a deeper understanding of the protein interactions underlying NPQ dynamics will be highly valuable in finding new ways to improve plant and microalgal productivity.

## 5 CONCLUSIONS

LHCSR‐ and STT7‐mediated NPQ processes (qE and qT) are known to underly the photoprotective response of the microalga *Chlamydomonas*. Here, we have applied a new method to disentangle the involvement of qE and qT in real time by exposing intact algal cells to repetitive cycles of high light and darkness alternating at different timescales. While both qE‐ and qT‐type responses are present during all light fluctuations, LHCSR‐dependent qE plays a larger role in the beginning of light fluctuations and during the HL periods. The contribution of STT7‐dependent qT became more pronounced upon longer exposure to fluctuating light and especially during the dark periods of light fluctuations. Over the long term, rapid light fluctuations reduced the growth of mutants impaired in LHCSRs, demonstrating the importance of LHCSR proteins during abrupt changes in light intensity. Overall, our work shows that a cooperativity between LHCSR proteins and STT7 constitutes an important regulatory feature of the photoprotective response in *Chlamydomonas*, likely mediated by phosphorylation of LHCSR3 by STT7. These findings provide a foundation for disentangling and modelling how the diverse molecular mechanisms involved in plant and microalgal acclimation to light fluctuations interact and enable robust photosynthesis in nature. We envision that further knowledge of the response of photosynthetic mechanisms to various timescales of light fluctuations will open new avenues for building a strong understanding of how photosynthetic organisms respond to complex light fluctuations.

## AUTHOR CONTRIBUTIONS

Collin J. Steen, Adrien Burlacot and Graham R. Fleming designed the research; Collin J. Steen, Adrien Burlacot and Audrey H. Short performed research; Collin J. Steen, Adrien Burlacot, Audrey H. Short and Graham R. Fleming analyzed data; Collin J. Steen and Adrien Burlacot wrote the paper with inputs from Audrey H. Short, Krishna K. Niyogi and Graham R. Fleming.

## CONFLICT OF INTEREST

The authors declare no conflict of interest.

## Supporting information

Supporting information.

## Data Availability

All data needed to evaluate the conclusions in the paper are present in the paper and/or the Supporting Information Materials.

## References

[pce14372-bib-0001] Allen, J.F. (1992) Protein phosphorylation in regulation of photosynthesis. Biochimica et Biophysica Acta, Bioenergetics, 1098, 275–335. 10.1016/s0005-2728(09)91014-3 1310622

[pce14372-bib-0002] Allorent, G. , Tokutsu, R. , Roach, T. , Peers, G. , Cardol, P. , Girard‐Bascou, J. et al. (2013) A dual strategy to cope with high light in *Chlamydomonas reinhardtii* . The Plant Cell, 25, 545–557. 10.1105/tpc.112.108274 23424243 PMC3608777

[pce14372-bib-0003] Amarnath, K. , Zaks, J. , Park, S.D. , Niyogi, K.K. & Fleming, G.R. (2012) Fluorescence lifetime snapshots reveal two rapidly reversible mechanisms of photoprotection in live cells of *Chlamydomonas reinhardtii* . Proceedings of the National Academy of Science USA, 109, 8405–8410. 10.1073/pnas.1205303109 PMC3365229 22586081

[pce14372-bib-0004] Aro, E.‐M. , Virgin, I. & Andersson, B. (1993) Photoinhibition of photosystem II. inactivation, protein damage and turnover. Biochimica et Biophysica Acta, Bioenergetics, 1143, 113–134. 10.1016/0005-2728(93)90134-2 8318516

[pce14372-bib-0005] Ballottari, M. , Truong, T.B. , De Re, E. , Erickson, E. , Stella, G.R. , Fleming, G.R. et al. (2016) Identification of ph‐sensing sites in the light harvesting complex stress‐related 3 protein essential for triggering non‐photochemical quenching in *Chlamydomonas reinhardtii* . Journal of Biological Chemistry, 291, 7334–7346. 10.1074/jbc.M115.704601 26817847 PMC4817166

[pce14372-bib-0006] Barera, S. , Dall'Osto, L. & Bassi, R. (2021) Effect of lhcsr gene dosage on oxidative stress and light use efficiency by *Chlamydomonas reinhardtii* cultures. Journal of Biotechnology, 328, 12–22. 33434600 10.1016/j.jbiotec.2020.12.023

[pce14372-bib-0007] Bellafiore, S. , Barneche, F. , Peltier, G. & Rochaix, J.‐D. (2005) State transitions and light adaptation require chloroplast thylakoid protein kinase STN7. Nature, 433, 892–895. 10.1038/nature03286 15729347

[pce14372-bib-0008] Benedetti, M. , Vecchi, V. , Barera, S. & Dall'Osto, L. (2018) Biomass from microalgae: the potential of domestication towards sustainable biofactories. Microb. Cell Factory, 17, 173. 10.1186/s12934-018-1019-3 PMC6230293 30414618

[pce14372-bib-0009] Bergner, S.V. , Scholz, M. , Trompelt, K. , Barth, J. , Gäbelein, P. , Steinbeck, J. et al. (2015) STATE TRANSITION7‐dependent phosphorylation is modulated by changing environmental conditions, and its absence triggers remodeling of photosynthetic protein complexes. Plant Physiology, 168, 615–634. 10.1104/pp.15.00072 25858915 PMC4453777

[pce14372-bib-0010] Björkman, O. & Demmig, B. (1987) Photon yield of O_2_ evolution and chlorophyll fluorescence characteristics at 77 K among vascular plants of diverse origins. Plantae, 170, 489–504. 10.1007/BF00402983 24233012

[pce14372-bib-0011] Bonaventura, C. & Myers, J. (1969) Fluorescence and oxygen evolution from *Chlorella pyrenoidosa* . Biochimica et Biophysica Acta, Bioenergetics, 189, 366–383. 10.1016/0005-2728(69)90168-6 5370012

[pce14372-bib-0012] Bonente, G. , Ballottari, M. , Truong, T.B. , Morosinotto, T. , Ahn, T.K. , Fleming, G.R. et al. (2011) Analysis of LhcSR3, a protein essential for feedback de‐excitation in the Green alga *Chlamydomonas reinhardtii* . PLoS Biology, 9, e1000577. 10.1371/journal.pbio.1000577 21267060 PMC3022525

[pce14372-bib-0013] Briantais, J.M. , Vernotte, C. , Picaud, M. & Krause, G.H. (1979) A quantitative study of the slow decline of chlorophyll a fluorescence in isolated chloroplasts. Biochimica et Biophysica Acta, Bioenergetics, 548, 128–138. 10.1016/0005-2728(79)90193-2 486438

[pce14372-bib-0014] Bru, P. , Steen, C.J. , Park, S. , Amstutz, C.L. , Sylak‐Glassman, E.J. , Lam, L. , Fekete A. , Mueller, M. J. , Longoni, F. , Fleming, G. R. , Niyogi, K. K. , & Malnoë, A . (2021) An energy‐dissipative state of the major antenna complex of plants. BioRxiv. 10.1101/2021.07.09.450705

[pce14372-bib-0015] Burlacot, A. , Dao, O. , Auroy, P. , Cuiné, S. , Li‐Beisson, Y. & Peltier, G. (2022) Alternative photosynthesis pathways drive the algal CO2 concentrating mechanism. Nature, 605, 366–371. 35477755 10.1038/s41586-022-04662-9

[pce14372-bib-0016] Cantrell, M. & Peers, G. (2017) A mutant of *Chlamydomonas* without LHCSR maintains high rates of photosynthesis, but has reduced cell division rates in sinusoidal light conditions. PLoS One, 12, e0179395. 10.1371/journal.pone.0179395 28644828 PMC5482440

[pce14372-bib-0017] Cardol, P. , Alric, J. , Girard‐Bascou, J. , Franck, F. , Wollman, F.A. & Finazzi, G. (2009) Impaired respiration discloses the physiological significance of state transitions in *chlamydomonas* . Proceedings of the National Academy of Sciences of the United States of America, 106, 15979–15984. 10.1073/pnas.0908111106 19805237 PMC2747229

[pce14372-bib-0018] Casper‐Lindley, C. & Björkman, O. (1996) Nigericin insensitive post‐illumination reduction in fluorescence yield in *Dunaliella tertiolecta* (chlorophyte). Photosynthesis Research, 50, 209–222. 10.1007/BF00033120 24271960

[pce14372-bib-0019] Correa‐Galvis, V. , Redekop, P. , Guan, K. , Griess, A. , Truong, T.B. , Wakao, S. et al. (2016) Photosystem II subunit PsbS is involved in the induction of LHCSR protein‐dependent energy dissipation in *Chlamydomonas reinhardtii* . Journal of Biological Chemistry, 291, 17478–17487. 10.1074/jbc.M116.737312 27358399 PMC5016143

[pce14372-bib-0020] Dall'Osto, L. , Caffarri, S. & Bassi, R. (2005) A mechanism of nonphotochemical energy dissipation, independent from PsbS, revealed by a conformational change in the antenna protein CP26. The Plant Cell, 17, 1217–1232. 10.1105/tpc.104.030601 15749754 PMC1087998

[pce14372-bib-0021] Depege, N. , Bellafiore, S. & Rochaix, J.D. (2003) Role of chloroplast protein kinase Stt7 in LHCII phosphorylation and state transition in *Chlamydomonas* . Science, 299, 1572–1575. 12624266 10.1126/science.1081397

[pce14372-bib-0022] Dinc, E. , Tian, L. , Roy, L.M. , Roth, R. , Goodenough, U. & Croce, R. (2016) LHCSR1 induces a fast and reversible ph‐dependent fluorescence quenching in LHCII in *Chlamydomonas reinhardtii* cells. Proceedings of the National Academy Sciences of the United States of America, 113, 7673–7678. 10.1073/pnas.1605380113 PMC4941471 27335457

[pce14372-bib-0023] Drop, B. , Webber‐Birungi, M. , Yadav, S.K.N. , Filipowicz‐Szymanska, A. , Fusetti, F. , Boekema, E.J. et al. (2014) Light‐harvesting complex II (LHCII) and its supramolecular organization in *Chlamydomonas reinhardtii* . Biochimica et Biophysica Acta, Bioenergetics, 1837, 63–72. 10.1016/j.bbabio.2013.07.012 23933017

[pce14372-bib-0024] Erickson, E. , Wakao, S. & Niyogi, K.K. (2015) Light stress and photoprotection in *Chlamydomonas reinhardtii* . The Plant Journal, 82, 449–465. 10.1111/tpj.12825 25758978

[pce14372-bib-0025] Fei, C. , Wilson, A.T. , Mangan, N.M. , Wingreen, N.S. & Jonikas, M.C. (2022) Modelling the pyrenoid‐based CO_2_‐concentrating mechanism provides insights into its operating principles and a roadmap for its engineering into crops. Nat. Plants, 8, 583–595. 10.1038/s41477-022-01153-7 35596080 PMC9122830

[pce14372-bib-0026] Forti, G. & Caldiroli, G. (2005) State transitions in *Chlamydomonas reinhardtii*. The role of the mehler reaction in state 2‐to‐state 1 transition. Plant Physiology, 137, 492–499. 10.1104/pp.104.048256 15591440 PMC1065350

[pce14372-bib-0027] Gao, S. , Pinnola, A. , Zhou, L. , Zheng, Z. , Li, Z. & Bassi, R. (2022) Light‐harvesting complex stress‐related proteins play crucial roles in the acclimation of *Physcomitrella patens* under fluctuating light conditions. Photosynthesis Research, 151, 1–15. 10.1007/s11120-021-00874- 34468919

[pce14372-bib-0028] Girolomoni, L. , Cazzaniga, S. , Pinnola, A. , Perozeni, F. , Ballottari, M. & Bassi, R. (2019) LHCSR3 is a nonphotochemical quencher of both photosystems in *Chlamydomonas reinhardtii* . Proceedings of the National Academy Sciences of the United States of America, 116, 4212–4217. 10.1073/pnas.1809812116 PMC6410775 30782831

[pce14372-bib-0029] Goral, T.K. , Johnson, M.P. , Duffy, C.D.P. , Brain, A.P.R. , Ruban, A.V. & Mullineaux, C.W. (2012) Light‐harvesting antenna composition controls the macrostructure and dynamics of thylakoid membranes in *Arabidopsis* . The Plant Journal, 69, 289–301. 21919982 10.1111/j.1365-313X.2011.04790.x

[pce14372-bib-0030] Graham, P.J. , Nguyen, B. , Burdyny, T. & Sinton, D. (2017) A penalty on photosynthetic growth in fluctuating light. Scientific Reports, 7, 12513. 10.1038/s41598-017-12923-1 28970553 PMC5624943

[pce14372-bib-0031] Huang, Z. , Shen, L. , Wang, W. , Mao, Z. , Yi, X. , Kuang, T. et al. (2021) Structure of photosystem I‐LHCI‐LHCII from the Green alga *Chlamydomonas reinhardtii* in state 2. Nature Communication, 12, 1100. 10.1038/s41467-021-21362-6 PMC7889890 33597543

[pce14372-bib-0032] Iwai, M. , Takizawa, K. , Tokutsu, R. , Okamuro, A. , Takahashi, Y. & Minagawa, J. (2010) Isolation of the elusive supercomplex that drives cyclic electron flow in photosynthesis. Nature, 464, 1210–U1134. 10.1038/nature08885 20364124

[pce14372-bib-0033] Iwai, M. , Yokono, M. , Inada, N. & Minagawa, J. (2010) Live‐cell imaging of photosystem II antenna dissociation during state transitions. Proceedings of the National Academy of Sciences of the United States of America, 107, 2337–2342. 10.1073/pnas.0908808107 20080575 PMC2836652

[pce14372-bib-0034] Kawakami, K. , Tokutsu, R. , Kim, E. & Minagawa, J. (2019) Four distinct trimeric forms of light‐harvesting complex II isolated from the Green alga *Chlamydomonas reinhardtii* . Photosynthesis Research, 142, 195–201. 10.1007/s11120-019-00669-y 31493286

[pce14372-bib-0035] Khorobrykh, S. , Havurinne, V. , Mattila, H. & Tyystjärvi, E. (2020) Oxygen and ROS in photosynthesis. Plants, 9, 91. 10.3390/plants9010091 PMC7020446 31936893

[pce14372-bib-0036] Klughammer, C. & Schreiber, U. (2008) Complementary PS II quantum yields calculated from simple fluorescence parameters measured by PAM fluorometry and the saturation pulse method. PAM Application Notes, 1, 201–247.

[pce14372-bib-0037] Kondo, T. , Pinnola, A. , Chen, W.J. , Dall'Osto, L. , Bassi, R. & Schlau‐Cohen, G.S. (2017) Single‐molecule spectroscopy of LHCSR1 protein dynamics identifies two distinct states responsible for multi‐timescale photosynthetic photoprotection. Nature Chemistry, 9, 772–778. 10.1038/nchem.2818 28754946

[pce14372-bib-0038] Kosuge, K. , Tokutsu, R. , Kim, E. , Akimoto, S. , Yokono, M. , Ueno, Y. et al. (2018) LHCSR1‐dependent fluorescence quenching is mediated by excitation energy transfer from LHCII to photosystem I in *Chlamydomonas reinhardtii* . Proceedings of the National Academy of Sciences of the United States of America, 115, 3722–3727. 10.1073/pnas.1720574115 29555769 PMC5889656

[pce14372-bib-0039] Kromdijk, J. , Głowacka, K. , Leonelli, L. , Gabilly, S.T. , Iwai, M. , Niyogi, K.K. et al. (2016) Improving photosynthesis and crop productivity by accelerating recovery from photoprotection. Science, 354, 857–861. 10.1126/science.aai8878 27856901

[pce14372-bib-0040] Lemeille, S. , Willig, A. , Depège‐Fargeix, N. , Delessert, C. , Bassi, R. & Rochaix, J.‐D. (2009) Analysis of the chloroplast protein kinase Stt7 during state transitions. PLoS Biology, 7, e1000045. 10.1371/journal.pbio.1000045 PMC2650728 19260761

[pce14372-bib-0041] Liguori, N. , Roy, L.M. , Opacic, M. , Durand, G. & Croce, R. (2013) Regulation of light harvesting in the Green alga *Chlamydomonas reinhardtii*: the c‐terminus of LHCSR is the knob of a dimmer switch. Journal of the American Chemical Society, 135, 18339–18342. 10.1021/ja4107463 24261574

[pce14372-bib-0042] Malnoë, A. , Schultink, A. , Shahrasbi, S. , Rumeau, D. , Havaux, M. & Niyogi, K.K. (2018) The plastid lipocalin LCNP is required for sustained photoprotective energy dissipation in *arabidopsis* . The Plant Cell, 30, 196–208. 10.1105/tpc.17.00536 29233855 PMC5810567

[pce14372-bib-0043] Minagawa, J. (2011) State transitions—the molecular remodeling of photosynthetic supercomplexes that controls energy flow in the chloroplast. Biochimica et Biophysica Acta, Bioenergetics, 1807, 897–905. 10.1016/j.bbabio.2010.11.005 21108925

[pce14372-bib-0044] Nagy, G. , Ünnep, R. , Zsiros, O. , Tokutsu, R. , Takizawa, K. , Porcar, L. et al. (2014) Chloroplast remodeling during state transitions in *Chlamydomonas reinhardtii* as revealed by noninvasive techniques in vivo. Proceedings of the National Academy of Sciences of the United States of America, 111, 5042–5047. 10.1073/pnas.1322494111 24639515 PMC3977285

[pce14372-bib-0045] Nawrocki, W.J. , Liu, X. & Croce, R. (2020) *Chlamydomonas reinhardtii* exhibits de facto constitutive NPQ capacity in physiologically relevant conditions. Plant Physiology, 182, 472–479. 10.1104/pp.19.00658 31653716 PMC6945880

[pce14372-bib-0046] Nawrocki, W.J. , Liu, X. , Raber, B. , Hu, C. , de Vitry, C. , Bennett, D.I.G. et al. (2021) Molecular origins of induction and loss of photoinhibition‐related energy dissipation qi. Science Advances , 7, 2021.2003.2010.434601 10.1126/sciadv.abj0055 PMC8694598 34936440

[pce14372-bib-0047] Nawrocki, W.J. , Santabarbara, S. , Mosebach, L. , Wollman, F.‐A. & Rappaport, F. (2016) State transitions redistribute rather than dissipate energy between the two photosystems in *Chlamydomonas* . Nature Plants, 2, 16031. 10.1038/nplants.2016.31 27249564

[pce14372-bib-0048] Nedbal, L. & Lazár, D. (2021) Photosynthesis dynamics and regulation sensed in the frequency domain. Plant Physiology, 187, 646–661. 10.1093/plphys/kiab317 34608969 PMC8491066

[pce14372-bib-0049] Nilkens, M. , Kress, E. , Lambrev, P. , Miloslavina, Y. , Müller, M. , Holzwarth, A.R. et al. (2010) Identification of a slowly inducible zeaxanthin‐dependent component of non‐photochemical quenching of chlorophyll fluorescence generated under steady‐state conditions in *Arabidopsis* . Biochimica et Biophysica Acta, Bioenergetics, 1797, 466–475. 10.1016/j.bbabio.2010.01.001 20067757

[pce14372-bib-0050] Niyogi, K.K. , Bjorkman, O. & Grossman, A.R. (1997) *Chlamydomonas* Xanthophyll cycle mutants identified by video imaging of chlorophyll fluorescence quenching. The Plant Cell, 9, 1369–1380. 10.1105/tpc.9.8.1369 12237386 PMC157004

[pce14372-bib-0051] Peers, G. , Truong, T.B. , Ostendorf, E. , Busch, A. , Elrad, D. , Grossman, A.R. et al. (2009) An ancient light‐harvesting protein is critical for the regulation of algal photosynthesis. Nature, 462, 518–521. 19940928 10.1038/nature08587

[pce14372-bib-0052] Perin, G. & Jones, P.R. (2019) Economic feasibility and long‐term sustainability criteria on the path to enable a transition from fossil fuels to biofuels. Current Opinion in Biotechnology, 57, 175–182. 31103911 10.1016/j.copbio.2019.04.004

[pce14372-bib-0053] Perozeni, F. , Beghini, G. , Cazzaniga, S. & Ballottari, M. (2020) *Chlamydomonas reinhardtii* LHCSR1 and LHCSR3 proteins involved in photoprotective non‐photochemical quenching have different quenching efficiency and different carotenoid affinity. Scientific Reports, 10, 21957. 10.1038/s41598-020-78985-w 33319824 PMC7738518

[pce14372-bib-0054] Pinnola, A. & Bassi, R. (2018) Molecular mechanisms involved in plant photoprotection. Biochemical Society Transactions, 46, 467–482. 10.1042/bst20170307 29666217

[pce14372-bib-0055] Rintamäki, E. , Martinsuo, P. , Pursiheimo, S. & Aro, E.‐M. (2000) Cooperative regulation of light‐harvesting complex II phosphorylation via the plastoquinol and ferredoxin‐thioredoxin system in chloroplasts. Proceedings of the National Academy of Sciences of the United States of America, 97, 11644–11649. 10.1073/pnas.180054297 11005828 PMC17254

[pce14372-bib-0056] Roach, T. (2020) LHCSR3‐Type NPQ prevents photoinhibition and slowed growth under fluctuating light in *Chlamydomonas reinhardtii* . Plants, 9, 1604. 10.3390/plants9111604 PMC7698959 33218177

[pce14372-bib-0057] Roach, T. & Na, C.S. (2017) LHCSR3 affects de‐coupling and re‐coupling of LHCII to PSII during state transitions in *Chlamydomonas reinhardtii* . Scientific Reports, 7, 43145. 10.1038/srep43145 28233792 PMC5324048

[pce14372-bib-0058] Roach, T. , Na, C.S. , Stöggl, W. & Krieger‐Liszkay, A. (2020) The non‐photochemical quenching protein LHCSR3 prevents oxygen‐dependent photoinhibition in *Chlamydomonas reinhardtii* . Journal of Experimental Botany, 71, 2650–2660. 10.1093/jxb/eraa022 31943079 PMC7210768

[pce14372-bib-0059] Rochaix, J.‐D. & Bassi, R. (2019) LHC‐like proteins involved in stress responses and biogenesis/repair of the photosynthetic apparatus. Biochemical Journal, 476, 581–593. 10.1042/BCJ20180718 30765616

[pce14372-bib-0060] Semchonok, D.A. , Sathish Yadav, K.N. , Xu, P. , Drop, B. , Croce, R. & Boekema, E.J. (2017) Interaction between the photoprotective protein LHCSR3 and C2S2 photosystem II supercomplex in *Chlamydomonas reinhardtii* . Biochimica et Biophysica Acta, Bioenergetics, 1858, 379–385. 28257778 10.1016/j.bbabio.2017.02.015

[pce14372-bib-0061] Steen, C.J. , Morris, J.M. , Short, A.H. , Niyogi, K.K. & Fleming, G.R. (2020) Complex roles of PsbS and xanthophylls in the regulation of nonphotochemical quenching in *Arabidopsis thaliana* under fluctuating light. The Journal of Physical Chemistry B, 124, 10311–10325. 10.1021/acs.jpcb.0c06265 33166148

[pce14372-bib-0062] Sylak‐Glassman, E.J. , Malnoë, A. , De Re, E. , Brooks, M.D. , Fischer, A.L. , Niyogi, K.K. et al. (2014) Distinct roles of the photosystem II protein PsbS and zeaxanthin in the regulation of light harvesting in plants revealed by fluorescence lifetime snapshots. Proceedings of the National Academy of Sciences of the United States of America, 111, 17498–17503. 10.1073/pnas.1418317111 25422428 PMC4267351

[pce14372-bib-0063] Sylak‐Glassman, E.J. , Zaks, J. , Amarnath, K. , Leuenberger, M. & Fleming, G.R. (2016) Characterizing non‐photochemical quenching in leaves through fluorescence lifetime snapshots. Photosynthesis Research, 127, 69–76. 10.1007/s11120-015-0104-2 25762378

[pce14372-bib-0064] Tanaka, Y. , Adachi, S. & Yamori, W. (2019) Natural genetic variation of the photosynthetic induction response to fluctuating light environment. Current Opinion in Plant Biology, 49, 52–59. 31202005 10.1016/j.pbi.2019.04.010

[pce14372-bib-0065] Tian, L. , Dinc, E. & Croce, R. (2015) LHCII populations in different quenching states are present in the thylakoid membranes in a ratio that depends on the light conditions. The Journal of Physical Chemistry Letters, 6, 2339–2344. 10.1021/acs.jpclett.5b00944 26266614

[pce14372-bib-0066] Tian, L. , Nawrocki, W.J. , Liu, X. , Polukhina, I. , van Stokkum, I.H.M. & Croce, R. (2019) pH dependence, kinetics and light‐harvesting regulation of nonphotochemical quenching in *Chlamydomonas* . Proceedings of the National Academy of Sciences of the United States of America, 116, 8320–8325. 10.1073/pnas.1817796116 30962362 PMC6486713

[pce14372-bib-0067] Tibiletti, T. , Auroy, P. , Peltier, G. & Caffarri, S. (2016) *Chlamydomonas reinhardtii* PsbS protein is functional and accumulates rapidly and transiently under high light. Plant Physiology, 171, 2717–2730. 10.1104/pp.16.00572 27329221 PMC4972282

[pce14372-bib-0068] Troiano, J.M. , Perozeni, F. , Moya, R. , Zuliani, L. , Baek, K. , Jin, E. et al. (2021) Identification of distinct ph‐ and zeaxanthin‐dependent quenching in LHCSR3 from *Chlamydomonas reinhardtii* . eLife, 10, e60383. 10.7554/eLife.60383 33448262 PMC7864637

[pce14372-bib-0069] Truong, T.B. (2011) Investigating the role(s) of LHCSRs in *Chlamydomonas reinhardtii* . Berkeley: University of California.

[pce14372-bib-0070] Ünlü, C. , Drop, B. , Croce, R. & van Amerongen, H. (2014) State transitions in *Chlamydomonas reinhardtii* strongly modulate the functional size of photosystem II but not of photosystem I. Proceedings of the National Academy of Sciences of the United States of America, 111, 3460–3465. 10.1073/pnas.1319164111 24550508 PMC3948275

[pce14372-bib-0071] Vecchi, V. , Barera, S. , Bassi, R. & Dall'Osto, L. (2020) Potential and challenges of improving photosynthesis in algae. Plants, 9, 67. 10.3390/plants9010067 PMC7020468 31947868

[pce14372-bib-0072] Vink, M. , Zer, H. , Alumot, N. , Gaathon, A. , Niyogi, K. & Herrmann, R.G. et al. (2004) Light‐modulated exposure of the light‐harvesting complex II (LHCII) to protein kinase(s) and state transition in *Chlamydomonas reinhardtii* xanthophyll mutants. Biochemistry, 43, 7824–7833. 10.1021/bi030267l 15196025

[pce14372-bib-0073] Wang, Y. , Burgess, S.J. , de Becker, E.M. & Long, S.P. (2020) Photosynthesis in the fleeting shadows: an overlooked opportunity for increasing crop productivity? The Plant Journal, 101, 874–884. 31908116 10.1111/tpj.14663 PMC7064922

[pce14372-bib-0074] Wehner, A. , Grasses, T. & Jahns, P. (2006) De‐epoxidation of violaxanthin in the minor antenna proteins of photosystem II, LHCB4, LHCB5, and LHCB6 J. Biological Chemistry, 281, 21924–21933. 10.1074/jbc.M602915200 16754673

[pce14372-bib-0075] Zakss, J. , Amarnath, K. , Kramer, D.M. , Niyogi, K.K. & Fleming, G.R. (2012) A kinetic model of rapidly reversible nonphotochemical quenching. Proceedings of the National Academy of Sciences of the United States of America, 109, 15757–15762. 10.1073/pnas.1211017109 22891305 PMC3465407

[pce14372-bib-0076] Zaks, J. , Amarnath, K. , Sylak‐Glassman, E.J. & Fleming, G.R. (2013) Models and measurements of energy‐dependent quenching. Photosynthesis Research, 116, 389–409. 10.1007/s11120-013-9857-7 23793348 PMC3824227

[pce14372-bib-0077] Zhang, X.J. , Fujita, Y. , Tokutsu, R. , Minagawa, J. , Ye, S. & Shibata, Y. (2021) High‐speed excitation‐spectral microscopy uncovers in situ rearrangement of light‐harvesting apparatus in Chlamydomonas during state transitions at submicron precision. Plant and Cell Physiology, 62, 872–882. 10.1093/pcp/pcab047 33822212

